# Physiological stress during cardiovascular magnetic resonance - handgrip exercise induced hemodynamic effects

**DOI:** 10.1186/1532-429X-14-S1-P229

**Published:** 2012-02-01

**Authors:** Florian von Knobelsdorff-Brenkenhoff, Matthias A Dieringer, Katharina Fuchs, Fabian Hezel, Wolfgang Renz, Thoralf Niendorf, Jeanette Schulz-Menger

**Affiliations:** 1 Working Group Cardiovascular MRI, Charite Medical University, Berlin, Germany; 2 Berlin Ultrahigh Field Facility, Max-Delbrueck-Center, Berlin, Germany; 3 Siemens Healthcare Sector, Erlangen, Germany; 4 Cardiology and Nephrology, HELIOS Klinikum Buch, Berlin, Germany

## Background

Pharmacological stress during cardiovascular magnetic resonance imaging (CMR) is limited by its non-physiological character and the risk of adverse events, whereas physical stress using bicycle exercise is challenging due to patients’ motion. This study tested the feasibility of handgrip exercise as an alternative and assessed its hemodynamic effects in volunteers.

## Methods

Twenty-nine volunteers (17 males, mean age 36±12years) underwent isometric handgrip stress testing using a CMR-compatible system (Sensory-Motor Systems Lab, Zurich, Switzerland) at 1/3 of the maximal voluntary contraction in a 3T scanner (Verio, Siemens, Germany). The actual force was presented to the volunteers on a screen via a mirror system to enable self-control. In all subjects, heart rate by electrocardiographic monitoring, and blood pressure (BP) by an arm cuff sphygmomanometer were repeatedly measured. The double product (heart rate x mean arterial BP) was used as an indicator of cardiac work. Left ventricular chamber quantification was done at baseline using steady-state free-precession cine imaging. In 11 volunteers, phase-contrast acquisitions were performed at the sinutubular level at rest and every minute of sustained handgrip exercise to quantify cardiac output.

## Results

Maximal voluntary contraction was 264±94N. Mean exercise duration was 7±2min. All flow measurements during exercise provided diagnostic image quality without motion artifacts. Heart rate, BP, stroke volume and double product increased in all subjects between rest and peak exercise (apart from n=1 with mildly decreasing BP). On average, heart rate increased from 65±3 to 80±14/min, systolic BP from 122±12 to 138±13mmHg, diastolic BP from 68±10 to 79±10mmHg, mean BP from 86±9 to 99±10mmHg, double product from 5487±1487 to 7763±1607mmHg/min and cardiac output from 5.4±0.8 to 7.4±1.1l/min (each p<0.001). Apart from males exhibiting a stronger increase of systolic BP compared to women (21±16 vs. 8±6mmHg; p=0.02), all other changes were independent of sex, age, left ventricular ejection fraction, enddiastolic volume index and mass index, as well as absolute handgrip force.

## Conclusions

Handgrip exercise testing during CMR is feasible and leads to considerable hemodynamic changes in healthy volunteers.

## Funding

The examinations were in part supported by the Else Kröner Fresenius Stiftung.

